# Autologous transplant for myeloma: when the old meets the new

**DOI:** 10.18632/oncotarget.21605

**Published:** 2017-10-08

**Authors:** Francesca Gay, Mariella Genuardi, Mario Boccadoro

**Affiliations:** Francesca Gay: Myeloma Unit, Division of Hematology, University of Torino, Azienda Ospedaliero-Universitaria Città della Salute e della Scienza di Torino, Torino, Italy

**Keywords:** multiple myeloma, transplant, novel agents, proteasome inhibitors, immunomodulatory drugs

Survival of multiple myeloma (MM) patients has dramatically improved in the last few decades thanks to the introduction of several novel agents and to improved supportive care strategies. Drugs with different mechanisms of action based on disease biology, including immunomodulatory (IMIDs) agents, proteasome inhibitors (PIs) and, more recently, monoclonal antibodies (MoAbs), administered in a total outpatient setting, have proved to be highly effective and easily manageable. In this context, although autologous transplantation (ASCT) represents a major advance and a cornerstone in the treatment of MM, the “old” high-dose therapy (HDT) - with the classical chemotherapy toxicities (cytopenia, infections, mucositis, alopecia) - has become progressively less appealing. The continuing and rapid drug development has repeatedly questioned the role of upfront ASCT. Novel agent-based therapies have been included in the pre-ASCT induction and/or post-ASCT consolidation and maintenance, as well as evaluated as multidrug combinations in the non-ASCT setting. At present, there are four randomized phase III trials reporting an improved progression-free survival (PFS) with upfront ASCT in comparison with no ASCT (Table 1) [1-4]. Despite the expected increase in adverse events with HDT and ASCT, toxicities were manageable with the current supportive care measures and did not translate into an increase in toxic deaths. Interestingly, the PFS advantage with ASCT was consistent in most of the analyzed subgroups. In two of the four trials, ASCT led also to an increase in overall survival (OS). What do these results mean in the complex treatment paradigm of MM in 2017?

**Table 1:** Main phase 3 trials with upfront transplantation

Protocol/Treatment schema	Median follow-up	PFS	OS
GIMEMA RV-MM-PI-209 [1] Rd-MEL200x2 *vs* Rd-MPR	51.2 months	Median: 43.0 *vs* 22.4 months (HR 0.44; *P* < 0.001)	4-year: 81.6% *vs* 65.3% (HR 0.55; *P* = 0.02)
EMN441 [2] Rd-MEL200x2 *vs* Rd-CRD	52.0 months	Median: 43.3 *vs* 28.6 months (HR 0.40; *P* < 0.0001)	4-year: 86% *vs* 73% (HR 0.42; *P* = 0.004)
IFM2009 [3] VRD-MEL200-VRD *vs* VRD	44 *vs* 43 months	Median: 50 *vs* 36 months (HR 0.65; *P* < 0.001)	4-year: 81% *vs* 82% (HR 1.16; *P* = 0.87)
EMN02 [4] VCD-MEL200 *vs* VCD-VMP	31.6 months	3-year: 65% *vs* 57.1% (HR 0.73; *P* = 0.001)	3-year: 86.3% *vs* 84.6% (HR 0.98; *P* = 0.899)

Rd, lenalidomide plus low-dose dexamethasone; MEL200, melphalan 200 mg/m2; MPR, melphalan-prednisone-lenalidomide; CRD, cyclophosphamide-lenalidomide-dexamethasone; VRD, bortezomib-lenalidomide-dexamethasone; VCD, bortezomib-cyclophosphamide-dexamethasone; VMP, bortezomib-melphalan-prednisone; PFS, progression-free survival; OS, overall survival; HR, hazard ratio.

First, it is clear that upfront ASCT still remains the standard of care in the context of a sequential approach including a pre-transplant induction and a post-transplant consolidation/maintenance with novel drugs. Its role has been confirmed regardless of the combination evaluated as comparator: ASCT led to superior PFS not only when compared with oral chemotherapy plus lenalidomide or plus bortezomib, but also when the two drugs were used in association, with a potentially higher efficacy [1-4]. Whether this advantage can be maintained over time remains a crucial issue. Data from two trials comparing ASCT vs oral chemotherapy plus lenalidomide have shown the superiority of ASCT for both PFS and the long-term endpoints PFS2 and OS; whereas no differences in OS between the ASCT and the non-ASCT arms have been so far reported in the IFM2009 and EMN02 trials [5]. Many factors affect long-term outcome, and treatment administered at relapse is certainly a major determinant. Of course, the higher the efficacy of the non-transplant arm, the lower the survival benefit needed with salvage therapy to obtain an OS similar to patients receiving upfront ASCT. A pooled analysis of the two lenalidomide-based trials reported that only 53% of patients treated with chemotherapy plus lenalidomide did receive ASCT at first relapse, and that the outcome of patients rescued with ASCT was superior if compared with the outcome of those who received other therapies [5]. Despite a possible “selection bias” (patients receiving ASCT at relapse might have been in better clinical conditions than patients who did not), these results support the role of ASCT also in the relapse setting. Of note, in the IFM2009 trial, where up to 79% of patients treated with lenalidomide plus bortezomib upfront were rescued with ASCT at relapse, no differences in OS were noticed [3]. Taken together, these two findings strengthen the importance of including ASCT in the overall treatment strategy of MM patients in 2017.

Two other relevant points are worth further discussion, namely risk stratification and response to treatment. These are in fact two important factors to define treatment strategy in many cancers. Generally, in the setting of curable diseases, low-risk patients may be treated with less intensive therapy, whereas high-risk patients require more intensive regimens. Subgroup analyses of all the four trials, despite being based on smaller subsets of patients, showed a HR for PFS in favor of ASCT (the more intensive treatment) in high-risk but also in standard-risk patients [1-4]. In the pooled analysis of the two ASCT vs oral chemotherapy plus lenalidomide trials, where the advantage of ASCT was evident also for PFS2 and OS, the PFS2 and OS benefit was retained in both good and bad prognosis patients [5]. Response to treatment, and in particular minimal residual disease (MRD) negativity, is another important predictor of outcome [6]. Data from three of the four trials showed that ASCT led to a higher rate of MRD negativity [3,7,8]. Moreover, in the IFM 2009 trial, ASCT prolonged PFS regardless of MRD status [3].

Altogether these data suggest that in 2017 upfront ASCT is the first choice in all eligible patients, independently of patient prognosis and response achieved. The higher the efficacy of the combination, the less marked the superiority of ASCT, although the PFS advantage with ASCT still remains significant. Ongoing and forthcoming trials exploring newer non-transplant combinations including second-generation PIs, IMIDs and MoAbs, may still challenge the role of upfront ASCT. A better and more comprehensive definition of patient prognosis and a more sensitive evaluation of MRD should be introduced in clinical trials evaluating - in a randomized fashion - how to tailor treatment according to patient prognosis and response. Results of these trials could change again the treatment scenario of MM in the future, hopefully with a shift towards a more tailored approach.

## References

[1] Palumbo A (2014). N Engl J Med.

[2] Gay F (2015). Lancet Oncol.

[3] Attal M (2017). N Engl J Med.

[4] Cavo M (2016). ASH.

[5] Gay F (2017). Leukemia.

[6] Munshi NC (2017). JAMA Oncol.

[7] Oliva S (2017). Oncotarget.

[8] Oliva S (2017). EHA.

